# Successful surgical resection and reconstruction of scrotal elephantiasis

**DOI:** 10.1002/iju5.12245

**Published:** 2020-12-11

**Authors:** Yuto Hattori, Naoki Hayata, Kenji Nakamura, Takeshi Takahashi, Kenji Mitsumori, Hiroyuki Ohnishi

**Affiliations:** ^1^ Department of Urology Osaka Red Cross Hospital Osaka Japan

**Keywords:** elephantiasis, genitalia, lymphedema, reconstructive surgical procedures

## Abstract

**Introduction:**

An enlarged scrotum due to scrotal elephantiasis results in a poor quality of life. This condition is uncommon and challenging to manage for most urologists. We report a case of scrotal elephantiasis treated with resection and scrotal reconstruction.

**Case presentation:**

A 57‐year‐old man was referred to our hospital with a 6‐year history of scrotal swelling. The scrotum was 20 cm in diameter, stiff, and thick. He was diagnosed with chronic scrotal lymphedema and underwent scrotal resection. The skin and subcutaneous tissues of the scrotum were excised, and the suprapubic skin, which was stretched by the enlarged scrotum, was used for the scrotal reconstruction. The penis was pulled out from this hole in the skin.

**Conclusion:**

Utilizing suprapubic skin flap for scrotal reconstruction is an effective treatment for scrotal elephantiasis that can result in functionally and cosmetically successful outcomes.

**Keynote message**

Scrotal elephantiasis can cause a marked reduction in the quality of life. Although conservative treatment can be used in early diagnosed cases, surgical intervention remains the most efficient approach for scrotal enlargement. We report a case of scrotal elephantiasis that was successfully treated with scrotal resection followed by reconstruction. This method utilized the redundant suprapubic skin to reconstruct the scrotum with excellent functional and cosmetic outcomes. No recurrence or complications were observed after 5 months.

## Introduction

Massive localized lymphedema of the scrotum is a rare disease that causes a reduction in the quality of life. For most urologists, managing this rare disease can be challenging. In general, the conservative treatment of patients with scrotal elephantiasis is inadequate and they eventually require surgical intervention. We report a case of scrotal elephantiasis that was successfully treated with resection and reconstruction with improved functional and cosmetic outcomes.

## Case presentation

A 57‐year‐old man was referred to our hospital with a 6‐year history of scrotal swelling. He underwent lymphovenous anastomosis 4 years prior; however, the scrotal size had been gradually increasing without any improvement.

He was obese with a body mass index of 35.3 kg/m^2^. His medical history included only well‐controlled hypertension and cerebral hemorrhage. On examination, his scrotum was swollen with a 20 cm maximum diameter and was stiff and thick. The penis was completely buried and impalpable. No swelling was observed in the other parts, including the lower limbs. Computed tomography revealed a uniform thickening of the subcutaneous layer of the scrotum, with no inguinal lymph node swelling.

**Fig. 1:**
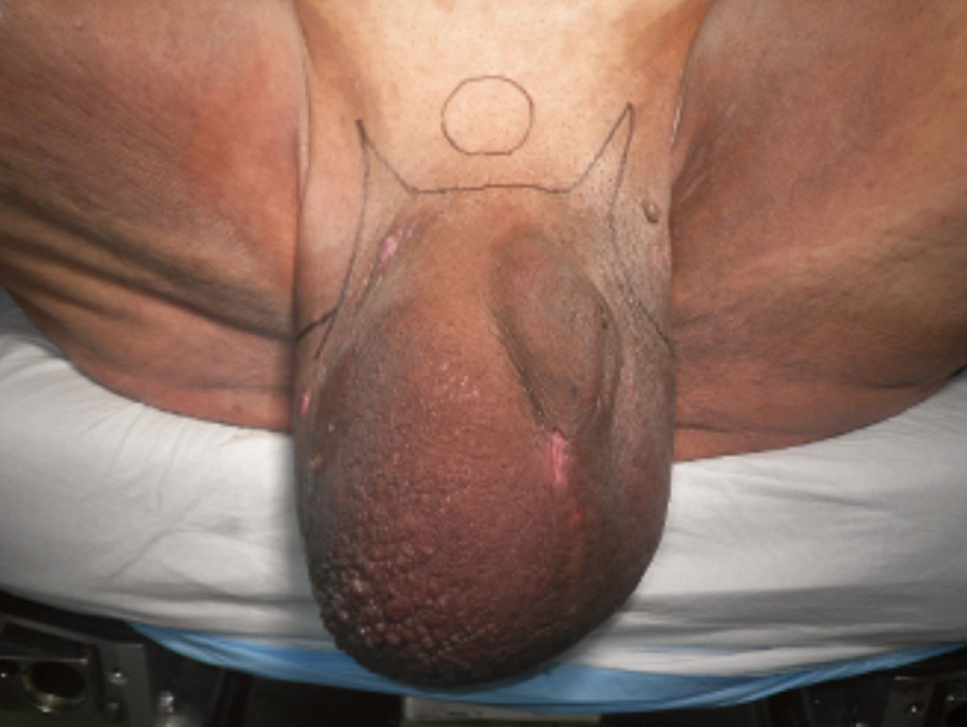
Preoperative appearance and design of the skin incision.

**Fig. 2:**
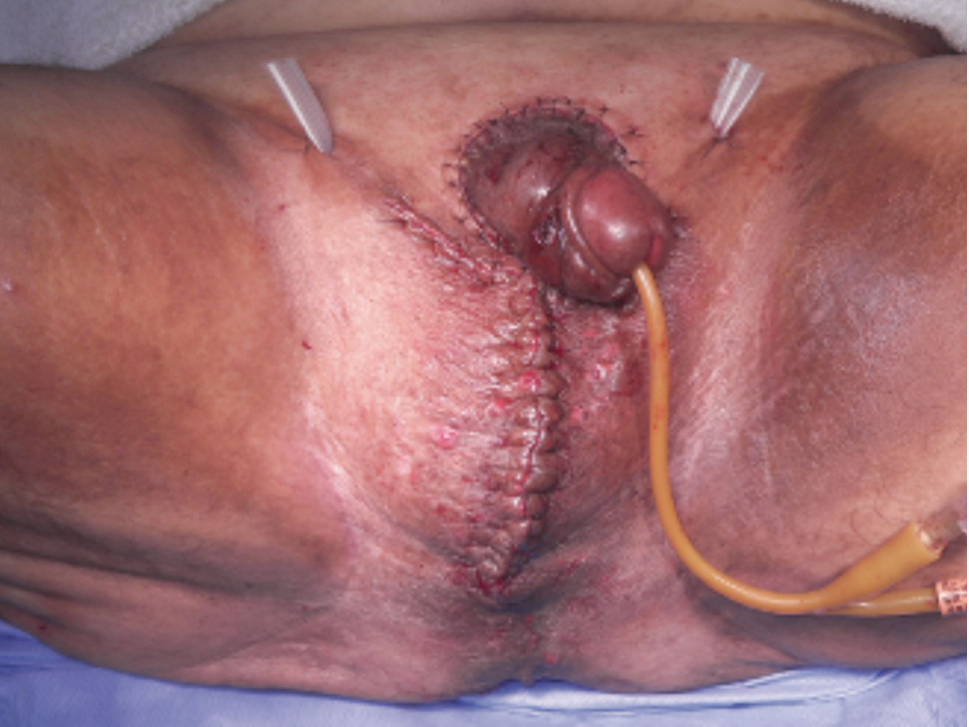
Postoperative result.

Secondary skin changes extended to the lateral side of the scrotum, which was deemed inappropriate for scrotal reconstruction. Furthermore, redundant suprapubic skin that was stretched by the enlarged scrotum was noted and we decided to use it to reconstruct the scrotum. A hole was created in this skin to deliver the penis (Fig. 1). Scrotal skin incision and subcutaneous dissection were performed to expose the tunica vaginalis and spermatic cord. After preserving these structures, the remaining mass was excised. The penis had no edematous changes, although phimosis was observed; thus, a dorsal slit procedure was also performed. The weight of the excised mass was 847 g (Fig. 2). Microscopic findings of the specimen revealed the proliferation of smooth muscle and fibrous tissue with inflammatory cell infiltration.

**Fig. 3:**
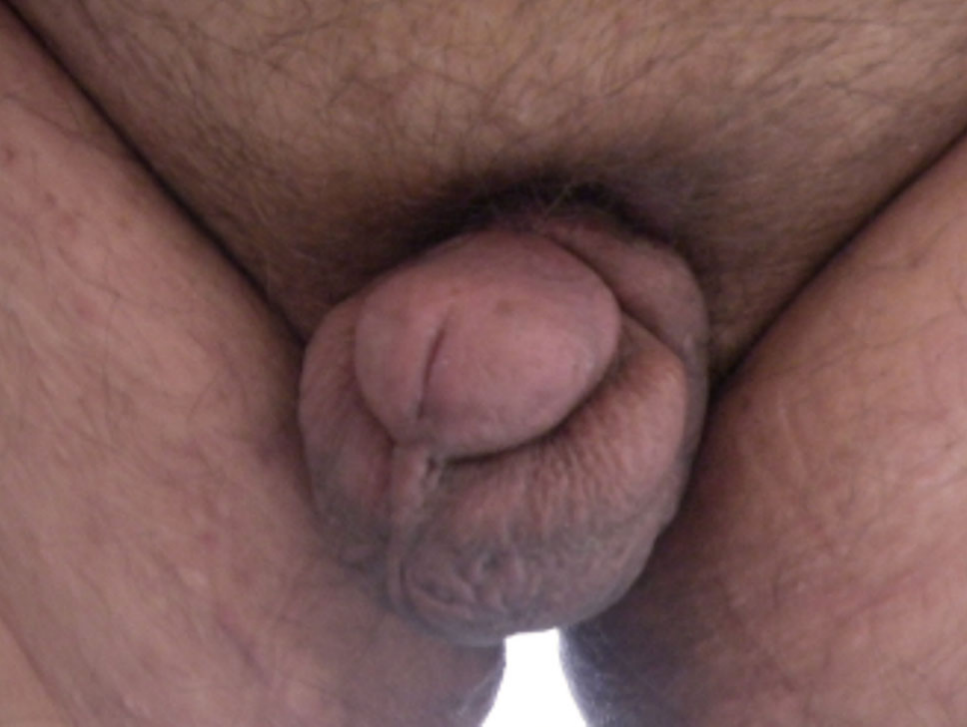
Findings 5 months postoperatively.

Penile edema appeared postoperatively, although it gradually improved except for the distal area to the dorsal incision. Wound breakdown occurred owing to the hematoma around the root of the penis, and it was treated with split‐thickness skin grafting. Postoperative findings showed that the patient was successfully treated with improved functional and cosmetic outcomes, and no recurrence or complications were seen after 5 months (Fig. 3).

## Discussion

Filariasis is a well‐known cause of elephantiasis; however, other causes exist, such as neoplastic, infectious, granulomatous, reactive, disorders of fluid balance, and idiopathic.^1^ Idiopathic cause is associated with obesity, and our case seems to be such.

An enlarged scrotum causes difficulty in performing the activities of daily living, such as walking, urination, sexual activity, and hygiene practices. Although compression and elevation have been attempted as conservative management, they could be only effective in early diagnosed cases. Similarly, lymphovenous anastomosis was inadequate and meaningless when an irreversible pathological change had already occurred. In scrotal elephantiasis classified as stage III lymphedema based on the International Society of Lymphology classification, surgical resection is the only effective approach to treat such condition.^1^, ^2^, ^3^

Genital lymphedema affects only the penoscrotal skin and subcutaneous tissues and does not reach the external spermatic fascia or the Buck’s fascia. The testis and inner preputial skin can be typically preserved owing to different lymphatic flow.^2^ A hydrocele is occasionally seen, and hydrocelectomy is also performed in such cases.^4^ The basic strategy for this type of surgery is to initially identify and preserve the spermatic cord and testis and subsequently remove the residual mass followed by scrotal reconstruction. Skin incision on the external inguinal ring or midline of the scrotum is reported as one of the methods to safely locate the spermatic cord and testis.^1^, ^5^ Preoperative urethral catheterization helps in identifying the urethra and preventing its injury, although it is frequently challenging due to the buried penis.

Several methods have been reported for scrotal resection and reconstruction; however, it is preferable to determine the surgical strategy for each case based on the degree of swelling and local skin condition. We previously treated a scrotal elephantiasis case with scrotal resection followed by penile resurfacing using lateral scrotal skin flaps. Although the scrotal lymphedema was cured, the redundant suprapubic skin still covered his penis postoperatively. Since the patient experienced difficulties with self‐care of his genital area, the suprapubic region should be included when surgical intervention is planned. In the present case, we could successfully manage this challenge by using suprapubic skin flap for scrotal reconstruction. It is believed to be a better procedure for patients with a buried penis. Since this problem is hardly recognized in the supine position, the patients should be preoperatively assessed in a standing or sitting position.

The necessity of penile skin resection is controversial. A previous report recommended penile skin removal in the case of incision involving around the penile base even if it looks healthy to prevent a postoperative edematous change.^5^ According to our case, the postoperative penile edema seems to be transient, and it is possible to achieve adequately satisfying outcomes without penile skin resection.

Furthermore, previous reports show that the incidence of complications such as infection, wound destruction, and recurrence is not low.^4^, ^5^ In addition, the effect of the neoscrotal environment on spermatogenesis is unknown. Therefore, it is desirable to establish a standard reconstruction procedure for elephantiasis by accumulating clinical cases.

## Conclusion

Surgical resection is necessary for scrotal elephantiasis. Utilizing the suprapubic skin flap for scrotal reconstruction can result in functionally and cosmetically excellent outcomes, especially for patients with a buried penis.

## Conflict of interest

The authors declare no conflict of interest.
